# Influence of corona poling on ZnO properties as *n*-type layer for optoelectronic devices

**DOI:** 10.1038/s41598-022-25984-8

**Published:** 2022-12-12

**Authors:** A. Magdy, A. El-Shaer, A. H. EL-Farrash, E. Salim

**Affiliations:** 1 Physics Department, Faculty of Science, Mansoura University, Mansoura, 35516 Egypt; 2 Physics Department, Faculty of Science, Kafrelsheikh University, Kafr El Sheikh, 33516 Egypt

**Keywords:** Materials science, Physics

## Abstract

Corona poling effects on optical and structural characteristics of zinc oxide (ZnO) thin films prepared by sol–gel spin coating technique were investigated. Atomic force microscope study showed the formation of pyramidal grains structure on the Corona-treated surface. The green–yellow photoluminescence peak centered at 2.36 eV and correlated to the antisite oxygen O_Zn_ defect, was found to decrease. X-ray diffraction patterns demonstrated that the Corona treatment enhanced the polycrystalline nature and increased the grain sizes of the ZnO thin films, which was also beneficial for electron transport. The role of the surface roughness of the ZnO thin film as electron transport layer in determining the photovoltaic effect of the inverted solar cells (ISCs) was examined by fabricating ISCs based on P3HT/PC_61_BM. The power conversion efficiency (PCE) obtained from these fabricated ISCs increased from 3.05 to 3.34%.

## Introduction

Organic solar cells (OSCs) have attracted a lot of interest during the last three decades due to their potential benefits in low-cost solar energy harvesting^1–3^. The most common type of OSCs is constructed based on a bulk heterojunction (BHJ) structure, with the photoactive layer composed of a blend of a donor (D)/acceptor sandwiched between a poly(3,4-ethylenedioxythiophene):poly(styrene sulfonate) (PEDOT:PSS)/indium tin oxide (ITO) anode and a low work function metal top cathode^4,5^. However, achieving high efficiency while maintaining long-term ambient air stability remains a critical problem for BHJ-OSCs. Inverted solar cells (ISCs) are one of the successful approaches for improving the stability and performance of BHJ-OSCs^6,7^. The development of ISCs is entirely dependent on the electrical and surface characteristics of cathode interface layers. As a result, the quest for an electron transport layer (ETL) leads to the use of a large number of metal oxides such as titanium oxide (TiO_x_)^8,9^, cesium carbonate (CsCO_3_)^10,11^, and zinc oxide (ZnO)^12^. Among all these ETL materials, ZnO is used more frequently due to its low work function, which enables the formation of an ohmic contact to be formed with the photoactive layer^13^. As well as ZnO possesses specific characteristics such as low cost, good air stability, nontoxicity, and high transparency in the visible/near-infrared spectral range^14^. There are several deposition techniques used to prepare the ZnO thin films such as atomic layer deposition (ALD)^15^, chemical vapor deposition (CVD)^16^, RF magnetron sputtering^17^, spray pyrolysis^18^, pulsed-laser deposition^19^, electrochemical deposition^20^, and sol–gel spin coating technique^21–23^. The sol–gel method offers the possibility of preparing thin film-supported nano-sized particles^24^, excellent control of the stoichiometry, and easy modification of film composition^25^.

In the present study, the influence of Corona poling treatment on the prepared ZnO thin film by the sol–gel method was investigated using atomic force microscope (AFM), UV–Vis absorption, photoluminescence (PL) spectroscopy, and X-ray diffraction (XRD). In addition, the photovoltaic performance of fabricated ISCs using ITO/ZnO/P3HT:PC_61_BM/MoO_3_/Ag architecture was presented. Furthermore, the comparative performance of these devices with and without Corona poling of ZnO thin films has been studied.

## Experimental details

### Materials

Zinc acetate dihydrate “Zn(CH_3_COO)2·2H_2_O” (99.9% purity), 2-methoxyethanol (99.8% purity), ethanolamine (99.5% purity), and 1,2-Dichlorobenzene (anhydrous, 99%) (DCB) were purchased from Sigma-Aldrich. Poly(3hexylthiophene) (P3HT) with 91–94% regio-regularity, and [6,6]-phenyl C61-butyric acid methyl ester (PC_61_BM) were purchased from Ossila. Indium-tin-oxide (ITO) coated glass substrates with a sheet resistance of (15–20) Ω/sq were obtained from Lumtec, Taiwan. All materials were used as-is, without further purification.

### Preparation of *n*-type ZnO sol–gel

**Figure 1:**
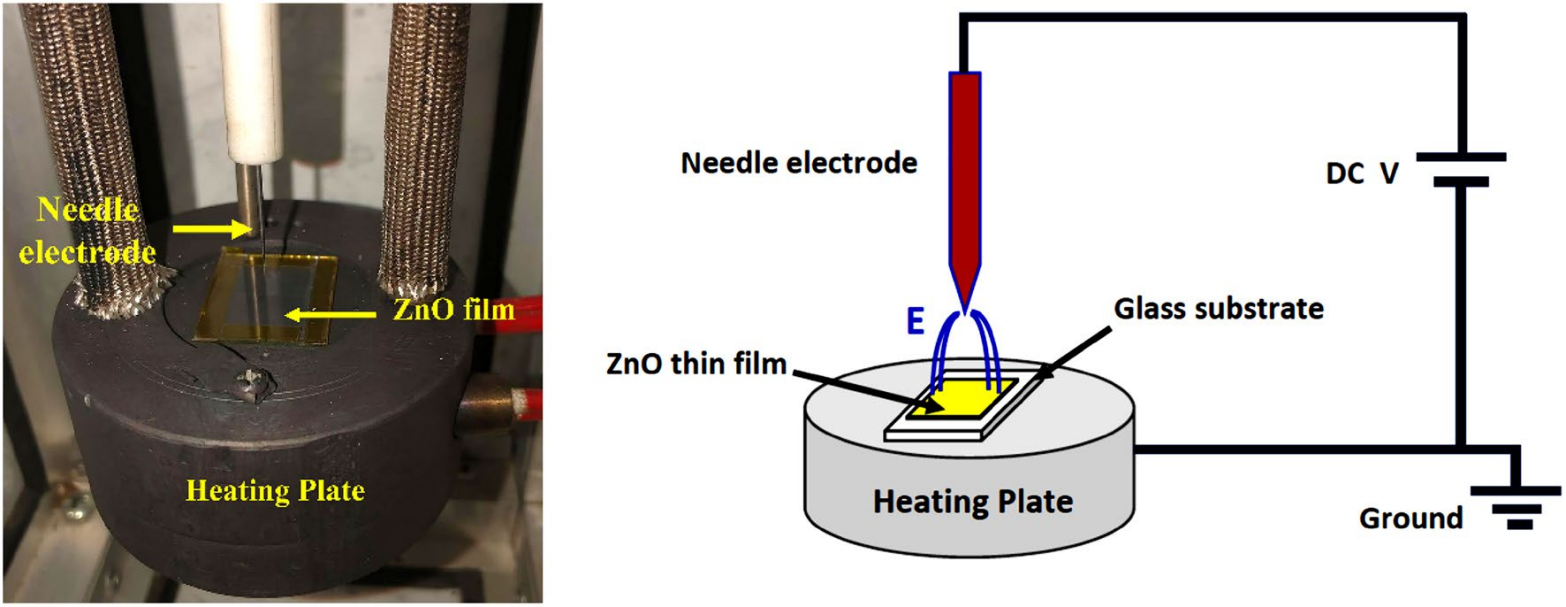
Schematic of Corona poling apparatus.

ZnO thin films were deposited on ITO-coated glass substrates using sol–gel processing method. The ITO substrates were sequentially washed by ultrasonication in a detergent, distilled water, isopropyl alcohol, and acetone for 10 min each. The washed and dried substrates were immediately transferred to oxygen plasma cleaner for 5 min. The zinc precursor was prepared by dissolving 0.5 g zinc acetate dihydrate in 5 ml 2-methoxyethanol with 0.14 mg ethanolamine (as a stabilizer) and stirred for 12 h under ambient air conditions. The thin films were deposited on plasma-washed ITO substrates using the spin-coating technique (3000 rpm, 40 s)^26,27^. The produced films were divided into two comparative films: the first film was annealed at 200 °C for 1 h. For the other film, it was annealed for 1 h at 200 °C under the Corona poling effect (6 kV DC applied voltage, 0.5 cm needle-sample distance) as shown in Fig. 1. After that, the applied voltage was maintained until the film reached room temperature. Both films were transferred into a glove box system for the next deposition steps.

### Device fabrication

**Figure 2:**
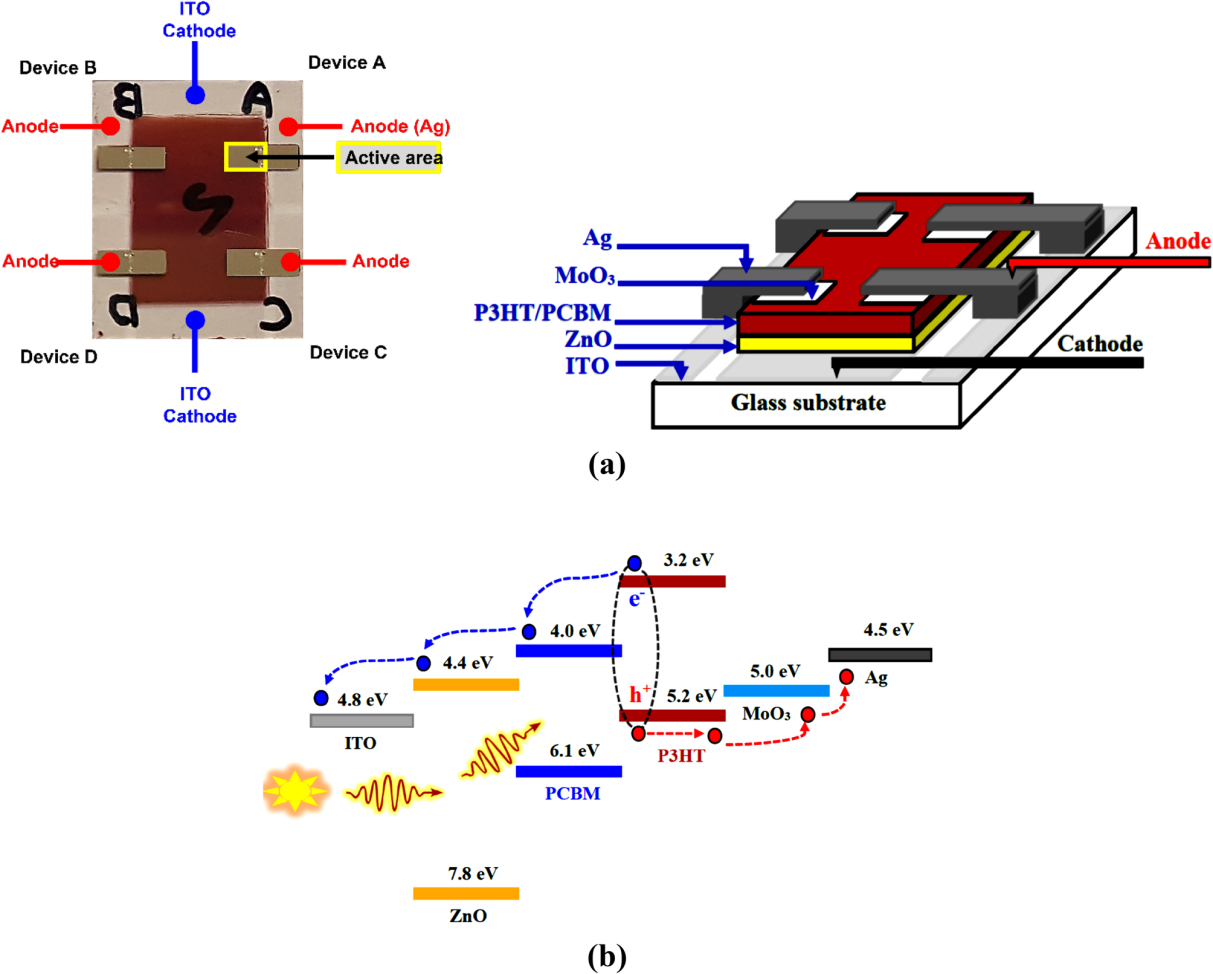
(**a**) The device representation of inverted BHJ organic solar cell, (**b**) Schematic description of energy level diagram of the inverted solar cell device.

A blend of 20 mg P3HT and 20 mg PC_61_BM was dissolved in 1 ml DCB with a weight ratio of 1:1 and stirred at 60 °C for 12 h. Two types of inverted organic solar devices were fabricated: the first device was deposited on the untreated ZnO layer and will be referred to hereafter as D1. The second device was fabricated on top of the Corona-treated ZnO layer and was named D2. A 0.45 µm tetrafluoroethylene (PTFE) filter was used to filter the stirred solution. The filtered solution of P3HT/PC_61_BM was spin-coated on top of both types of ZnO layers at 600 rpm for 1 min and then annealed at 100 °C for 10 min. Finally, MoO_3_ (7 nm) and Ag (100 nm) were thermally deposited through a shadow mask forming a device area of 0.06 cm^2^ for both devices. MoO_3_ essentially acts as a hole transport layer (HTL) and manifestly promotes the ohmic contact between the active layer and Ag anode for accelerating the hole extraction^28^. The inverted structure of both devices D1 and D2 was ITO/ZnO/P3HT:PC_61_BM/MoO_3_/Ag as shown in Fig. 2a.

### Characterization

The surface morphology of the ZnO thin films was examined using an atomic force microscope (flex AFM3). Contact mode was used during the scanning with a Nano surf C300 (version 3.5.0.31) software. The UV–visible absorption spectrum of the ZnO thin films was obtained by the JASCO (V-630) UV/Vis spectrophotometer. Photoluminescence (PL) setup (He-Cd laser, CW, 325 nm, Max.200mW, KIMMON KOHA CO., LTD.) was used for measuring the PL spectra of ZnO thin films. The X-ray diffraction (XRD) patterns have been recorded using a Shimadzu XRD-6000 X-ray diffractometer. The current–voltage measurements have been recorded under 100 mW/cm^2^ of AM 1.5 G irradiation using a computer-controlled Keithley 2400 source meter unit.

## Results and discussion

### Optical properties

**Figure 3:**
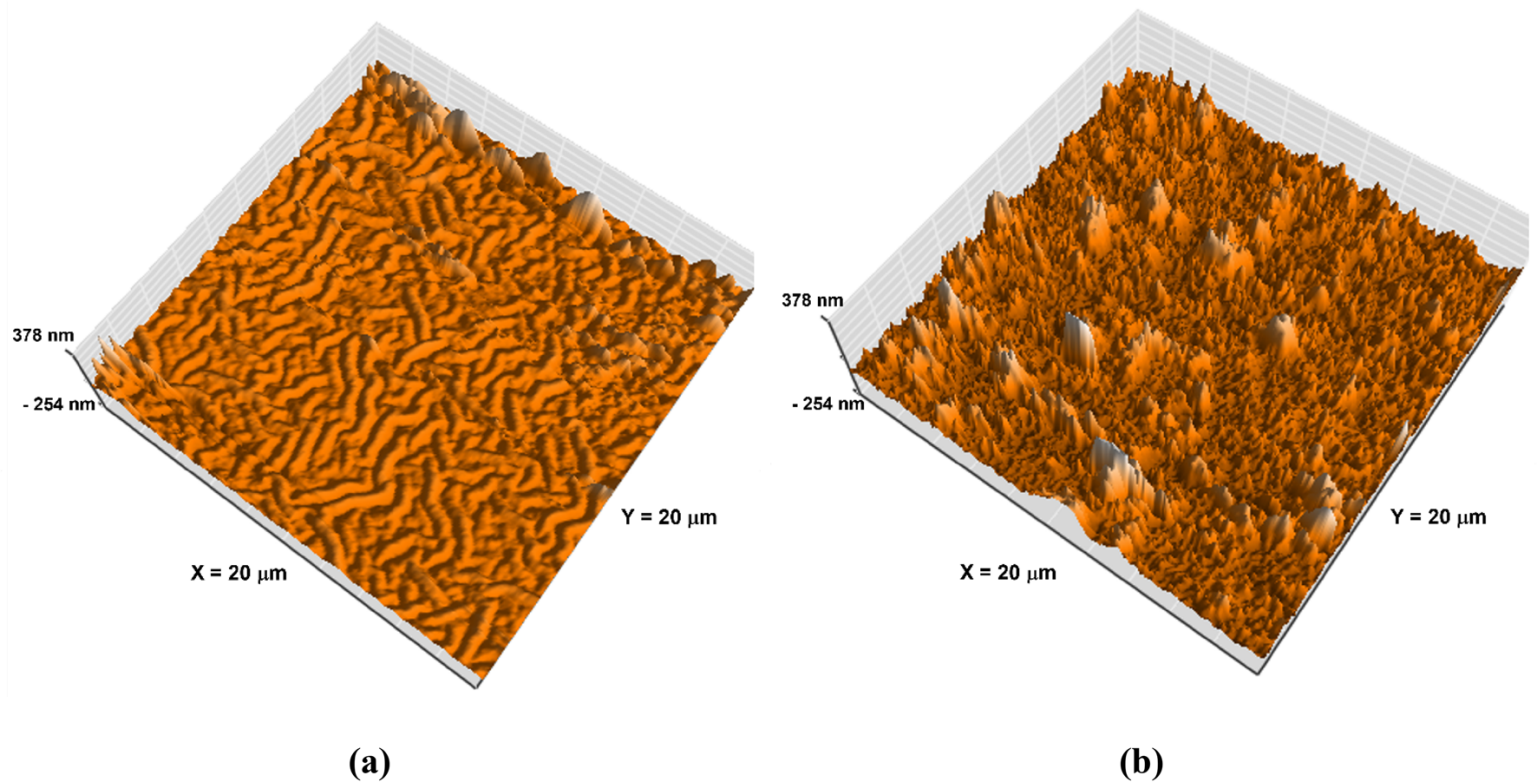
The 3D AFM images of the ZnO layer (**a**) annealed at 200 °C, and (**b**) annealed at 200 °C under applied 6 kV.

Figure 2B represents the highest occupied molecular orbital (HOMO) and lowest unoccupied molecular orbital (LUMO) energy levels of the fabricated device. To investigate the influence of Corona poling on the surface morphology of the ZnO film, AFM measurements were performed and are presented in Fig. 3. Upon close examination of the AFM images, it was observed that the surface of the untreated Corona ZnO film consists of elongated grains with a root mean square (RMS) roughness of 28.65 nm (Fig. 3a). However, the ZnO film after Corona treatment shows a pyramidal grains structure and exhibits a large RMS surface roughness of 66.16 nm as shown in Fig. 3b. Therefore, it is apparent that the Corona poling effect can significantly change the morphology of the surface. The increase in the surface roughness may effectively reduce the charge-transport distance and improve the photocurrent Jsc. In addition, the sharp nanoscale texture on the surface of the Corona-treated ZnO thin film may further enhance the electron extraction from the photoactive layer.

**Figure 4:**
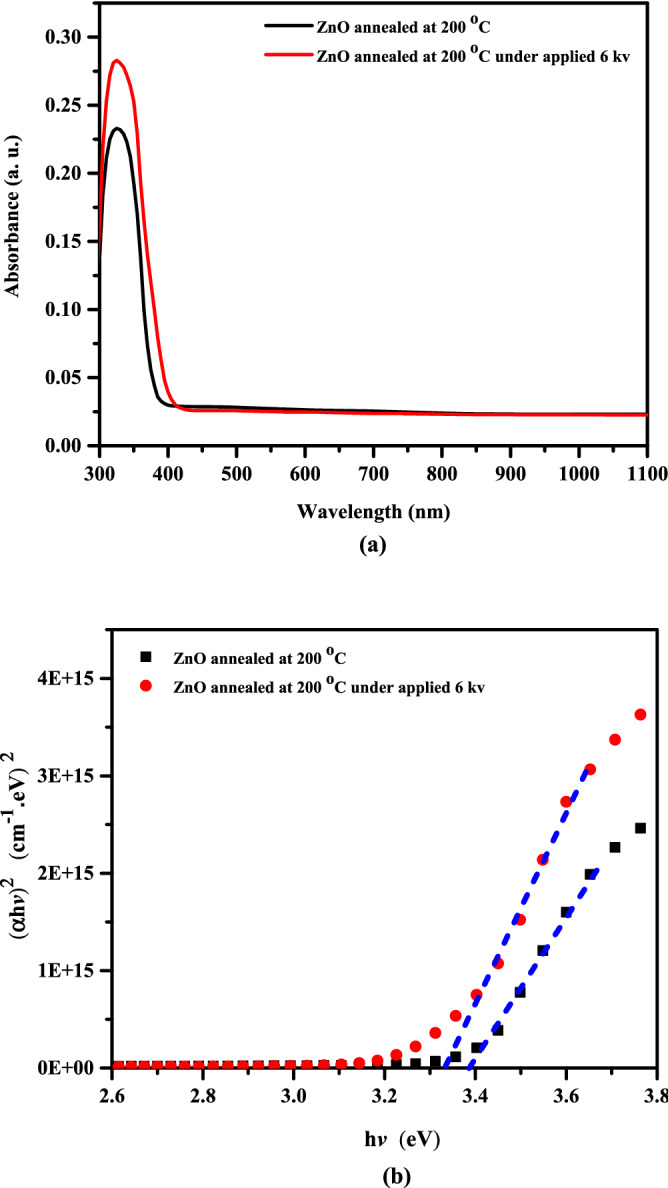
(**a**) Absorbance of ZnO thin films, (**b**) Plot of (αh*v*)^2^ versus hν for ZnO thin films.

The UV–visible absorbance of the ZnO thin films have been investigated in the wavelength range of 300–1100 nm as a function of Corona treatment. As can be seen from Fig. 4a, the wavelength of an excitonic absorption is about 325 nm. The existence of this excitonic peak indicates that the ZnO films have good structural quality. The treated ZnO film by 6 kV shows lower absorption in the visible range (400–700 nm). The value of optical direct-band gap (${\text{E}}_{{\text{g}}}^{{\text{d}}}$) of the ZnO films was obtained using the following relation by extrapolating the linear component of (αhυ)^2^ versus (hυ) plots^29,30^:

1 $$ \left( {\alpha {\text{h}}\upsilon } \right)2 = \beta ({\text{h}}\upsilon - {\text{E}}_{{\text{g}}}^{{\text{d}}} ) $$

where α is the absorption coefficient, β is a constant, and hυ is the photon energy. Figure 4b shows the (αhυ)^2^ versus (hυ) plots. The value of ${\text{E}}_{{\text{g}}}^{{\text{d}}}$ of the ZnO thin films was observed to decrease from 3.39 to 3.33 eV with the Corona treatment. This decrease in the optical band gap may be ascribed to an increase in grain size.

**Figure 5:**
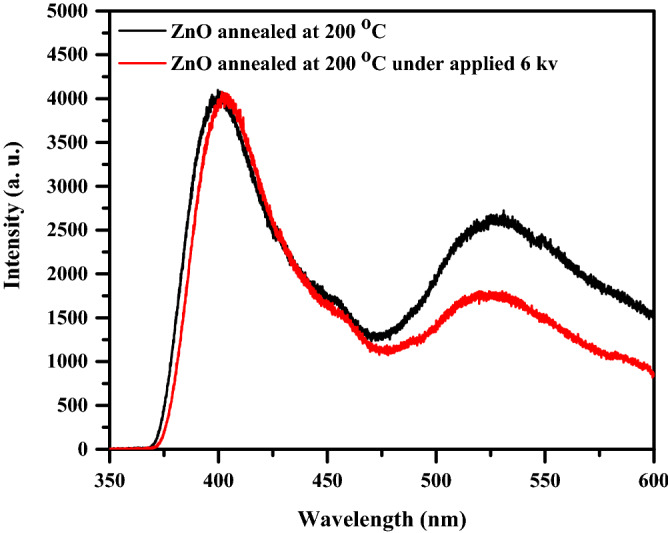
PL spectra of ZnO thin films.

**Figure 6:**
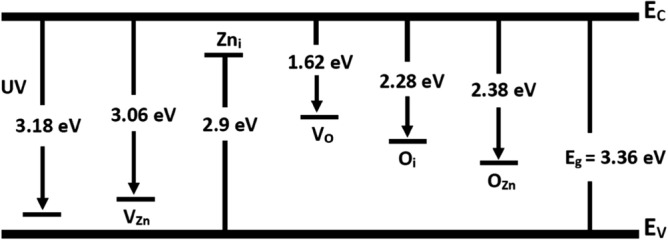
The calculated defect levels in ZnO film^32^.

The photoluminescence (PL) spectra of the prepared ZnO thin films measured at room temperature are plotted in Fig. 5. Two luminescence peaks are observed in both spectra of ZnO thin films, the first peak centered at 3.11 eV near the band edge and is assigned to free exciton emission^31^. The other peak is a broad green–yellow emission located at 2.36 eV, which may be due to the intrinsic defects in the ZnO thin films. There are five types of intrinsic defects in ZnO films; zinc vacancy V_Zn_, oxygen vacancy V_O_, interstitial zinc Zn_i_, interstitial oxygen O_i_, and antisite oxygen O_Zn_. Sun used the full-potential linear muffin-tin orbital technique to compute^32^ the energy levels of the intrinsic defects in ZnO film as shown in Fig. 6. The energy gap of 2.38 eV from the bottom of the conduction band to the O_Zn_ level matches the energy of the green–yellow emission seen in our spectra. That is, the green–yellow emission was caused mostly by O_Zn_ defects^33^. It is noted that a slight redshift in the UV emission is observed at 3.0 eV for the ZnO treated film by 6 kV. The redshift of the UV emission may be attributed to the increase in grain size. While the PL intensity associated with green–yellow emission has been found to decrease. This decrease in the green–yellow emission may be correlated to the decrease in the concentration of antisite defect O_Zn_ in ZnO thin film. We propose that the corona poling effect promotes more Zn_i_ and V_O_ resulting in low O_Zn_. The dependence of particle size on Corona treatment is consistent with the XRD pattern discussed below.

### Structural properties

**Figure 7:**
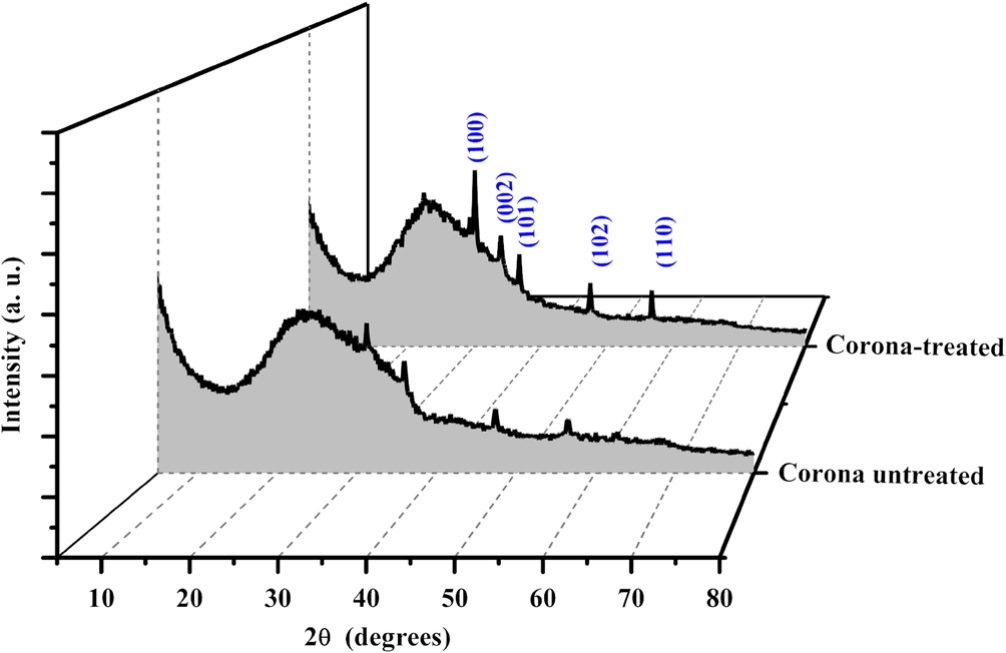
X-ray diffraction pattern of ZnO layers.

X-ray diffraction (XRD) has been used to investigate the influence of Corona poling treatment on the structure of the ZnO thin film as shown in Fig. 7. The diffraction peaks at 30.02°, 34.01°, 36.77°, 47.49°, and 56.77° corresponding to ZnO (100), (002), (101), (102), and (110) planes, respectively, were observed in both ZnO films^34^. It is found that the intensity of the peaks increased with the Corona-treated film. The enhanced intensity of the peaks indicates preferred orientation along the c-axis and indicates the polycrystalline nature of the ZnO film.

The crystallite sizes (D) of the films were estimated from the full-width at half-maximum (FWHM) of the diffraction peaks by the Debye-Scherrer equation as follows^35^:

2 $$ {\text{D}} = \frac{0.9 \lambda }{{\beta \cos \theta }} $$

where λ is the wavelength of the X-ray (λ = 0.154 nm), β is the broadening of the peak at FWHM, and θ is the Bragg angle of the peak. The estimated structural parameters are listed in Table 1. It is observed that the grain sizes increased with the impact of the Corona poling treatment.

**Table 1:** The XRD parameters of the ZnO thin films.

2 θ (°)	h k l	ZnO annealed at 200 °C		ZnO annealed at 200 °C under applied 6 kV	
		FWHM	D (nm)	FWHM	D (nm)
30.05	1 0 0	0.881	9.35	0.518	15.85
34.01	0 0 2	1.73	4.79	0.523	15.84
36.77	1 0 1	–	–	0.317	26.37
47.49	1 0 2	0.589	14.71	0.469	18.49
56.77	1 1 0	0.537	16.77	0.427	21.13

### Photovoltaic properties

**Figure 8:**
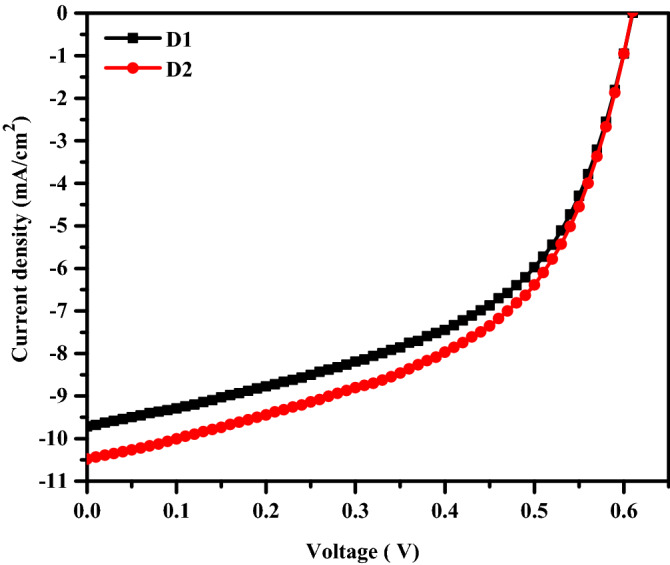
J–V characteristics curves of fabricated devices under AM 1.5

**Figure 9:**
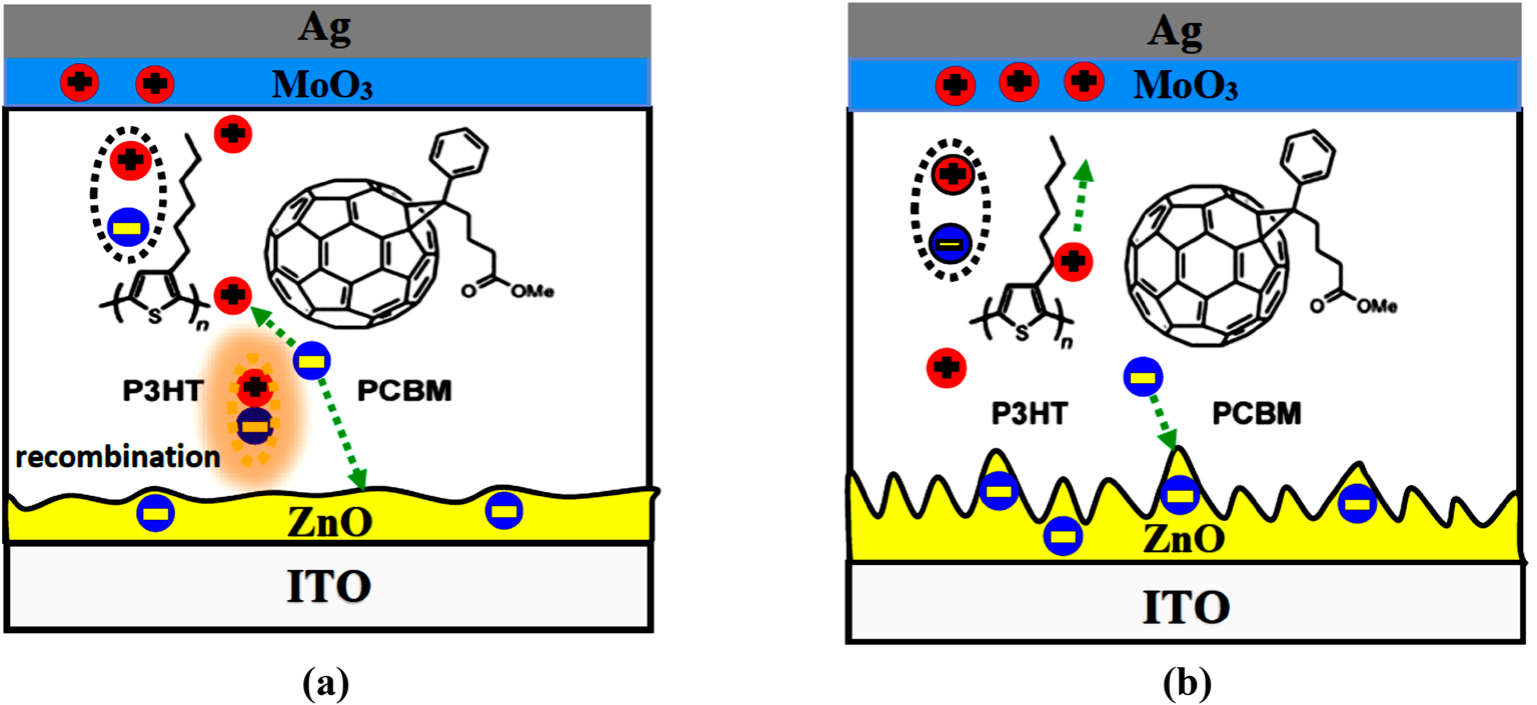
Schematic diagram of inverted bulk heterojunction solar cell deposited on top of (**a**) ZnO layer annealed at 200 °C and (**b**) ZnO layer annealed at 200 °C under applied 6 kV.

Next, using an ISC device design (see Fig. 2a), we examined the PV characteristics of the fabricated devices D1 and D2 that were deposited on top of the untreated and Corona-treated ZnO layer, respectively. Figure 8 shows representative current density versus voltage (J–V) characteristics of solar cell devices under 100 mW/cm^2^ of AM 1.5 G irradiation. The extracted PV data are listed in Table 2. The device D1 exhibits an open-circuit voltage (V_oc_) of 0.61 V, a short circuit current density (J_sc_) of 9.67 mA/cm^2^, a fill factor (FF) of 51.80%, and power conversion efficiency (PCE) of 3.05%. However, device D2 shows a V_oc_ of 0.61 V, a J_sc_ of 10.46 mA/cm^2^, a FF of 52.38%, and a PCE of 3.34%. It can be noticed that device D2 shows enhanced performance compared to D1. Both J_sc_ and PCE for D2 are enhanced dramatically while the V_oc_ of both devices does not change. The ZnO is widely recognized for its role as a hole blocking and electron-transporting layer. So, the enhanced performance of the device D2 is attributed to the increase in surface roughness of the treated ZnO layer which in turn increased the contact interfacial areas with the photoactive layer as clarified in Fig. 9a,b. Furthermore, the sharp nanoscale texture on the surface of the Corona-treated ZnO layer may provide more continuous electron transport pathways and facilitates excitons dissociation.

**Table 2:** Photovoltaic parameters extracted from the J–V curves in Fig. 7.

Device	V_OC_ (V)	J_SC_ (mA/cm^2^)	FF (%)	R_S_ (Ω cm^2^)	R_SH_ (kΩ cm^2^)	PCE (%)
D1	0.61	9.67 ± 0.05	51.80 ± 2	10.42	0.291	3.05 ± 0.1
D2	0.61	10.46 ± 0.04	52.38 ± 2	10.04	0.308	3.34 ± 0.08

## Conclusion

In summary, the influence of Corona poling treatment on structural, absorption, and PL spectra has been critically investigated in sol–gel-derived ZnO thin films on glass substrates. XRD pattern of ZnO thin film treated by Corona poling showed enhanced polycrystalline nature and larger grain sizes than untreated film. The larger the grain size, the fewer grain boundaries and surface traps. The absorption edge analysis revealed that the optical band gap energy decreases with the Corona treatment. A UV emission peak at 3.11 eV and a green–yellow emission peak at 2.36 eV are observed in the PL emission spectra. The intensity of the green-yellow peak due to the antisite oxygen O_zn_ defect decreased upon the Corona-poling effect. AFM images showed a sharp nanoscale texture with a RMS of 66.16 nm on the topography of the Corona-treated ZnO thin film. It is found that the J_sc_ was enhanced in the ISC device based on the Corona-treated ZnO layer, with a large ZnO/P3HT: PC_61_BM contact interfacial area that facilitates exciton separation.
